# Effect of a Dairy Cow’s Feeding System on the Flavor of Raw Milk: Indoor Feeding or Grazing

**DOI:** 10.3390/foods12091868

**Published:** 2023-04-30

**Authors:** Xuelu Chi, Ning Yuan, Yangdong Zhang, Nan Zheng, Huimin Liu

**Affiliations:** 1 Key Laboratory of Quality & Safety Control for Milk and Dairy Products of Ministry of Agriculture and Rural Affairs, Institute of Animal Sciences, Chinese Academy of Agricultural Sciences, Beijing 100193, China; chi_xl@163.com (X.C.);; 2 College of Animal Science, Xinjiang Agricultural University, Urumqi 830052, China

**Keywords:** grazing, indoor feeding, flavor, taste, raw milk

## Abstract

The flavor of fresh, raw milk is considered to be the key to maintaining the quality of dairy products, and is very crucial in affecting a consumer’s choice. To better understand the differences in flavor of fresh milk between feeding patterns, we conducted the following study. Twelve Holstein cows reared in pure grazing mode and twelve reared intensively in medium to large farms were selected from the Xinjiang Uygur Autonomous Regions at the same time, and the flavor of their raw milk was analyzed. Aroma profiles and taste attributes were assessed by electronic nose and electronic tongue, respectively, and volatile flavor compounds were characterized and quantified by Headspace-Solid Phase Microextraction/Gas Chromatography-Mass Spectrometry. Thirteen volatile compounds were identified in the indoor feeding pattern and 12 in the grazing; most of them overlapped. W1S, W2S and W5S were the main contributing sensors of the electronic nose for the overall assessment of the aroma profile. Raw milk from grazing had more intense astringency, bitterness, sourness and richness in taste compared to indoor feeding. Different dietary conditions may contribute to a variety of aroma profiles. Oxime-, methoxy-phenyl-, octadecanoic acid, furfural and dodecanoic acid were the key volatile flavor compounds of grazing. Meanwhile, raw milk from indoor feeding patterns was unique in 2-nonanone, heptanoic acid and n-decanoic acid. All three detection techniques were valid and feasible for differentiating raw milk in both feeding patterns, and the compounds were significantly correlated with the key sensors by correlation analysis. This study is promising for the future use of metabolic sources of volatile organic compounds to track and monitor animal feeding systems.

## 1. Introduction

Sensory attributes play a key role in determining consumer acceptability for dairy products. Compared with other dairy products, such as UHT milk, yoghurt, butter or cheese, the type and concentration of aroma compounds in fresh milk are very low [1]. The organoleptic characteristics of dairy products are directly influenced by the flavor of raw milk. Fresh cow’s milk has a distinctive yet subtle delicate flavor, which is easily affected. Potential factors in this study involved type of forage [2,3], patterns of feeding [4] and animal breeds [5,6]. In addition, process conditions [7,8,9,10], types of products [11], packing materials [12] and storage [13] were also key influencing factors.

For raw milk, the feeding pattern is a direct and critical element. Volatile flavor compounds derived from the diet that potentially transfer directly from forage or act as substrates eventually accumulate in milk [14]. Grazing systems are associated with increased product quality and greater global sustainability [15,16,17]. For consumers, the grazing model may signify more freedom, better health and pristine conditions. Therefore, they are increasingly interested in choosing dairy products produced from the milk of pasture-fed ruminants [16,18,19,20]. Previous studies have described the potential for terpenoids and carotenoids in fresh grass that affect milk flavor directly or indirectly [21]. In addition, the quality of milk in grazing patterns depends primarily on environmental factors that include biodiversity and the plant composition of grasslands [5]. Cows on pasture-based and TMR-based diets are distinguished from their milk by human sensory analysis, proximate analysis and volatile flavor compounds [4]. However, there were conflicting results on the effect of different forage types on milk flavour. For example, a more barny flavor in milk from grazing compared with total mixed ration (TMR) only was detected by a trained panel, but consumers did not detect the difference between them [22]. Flavor has been increasingly valued for applications such as geographical traceability, identification of forage differences, certification of organic products and classification of feeding types.

Electronic noses and electronic tongues as biomimetic designs can aid in increased sensitivity and selectivity. These two evaluation tools are effective methods in sensory evaluation, which avoid the potential risk of pathogenic microorganisms in raw milk instead of manual sensory evaluation [23]. E-nose is used as a non-destructive technical tool for monitoring microbial growth [24], identifying milk product brands [25,26,27], distinguishing between grazing and indoor feeding goat milk [28], and food traceability/authentication [18,29,30]. E-tongue is found to have considerable application in the detection of goat’s milk adulteration [31,32], the assessment of heat treatment [33], the differentiation of pasteurized milk brands [26] and the identification of microbiological species [29,34]. HS-SPME-GC-MS shows significant advantages in the analysis of food flavor compounds, and the technique is well-established for dairy analysis.

In this study, raw milk from Holstein cattle reared in two different feeding patterns (grazing and indoor feeding) was obtained simultaneously in the same area at the same time. The effects of grazing and indoor feeding on flavor in raw milk were assessed by HS-SPME/GC-MS combined with the advantages of intelligent sensory techniques. As a result, the aroma components and taste properties of raw milk in different feeding patterns are comprehensively explained, while the differences in flavor are analyzed. This study will provide a reference for raw milk quality control, and new insights for improving the sensory quality of dairy products and identifying raw milk from different feeding patterns.

## 2. Materials and Methods

### 2.1. Milk Sampling

All samples were collected from the Xinjiang Uyghur Autonomous Region, China, in the third week of December 2022. Twelve samples were collected from indoor feeding patterns with herd sizes > 300 cows and milked 2–3 times per day. The lactating cows were in parity 2, day in milk (140–175 d), and daily milk yield was around 25 kg. Another twelve samples from grazed feeding patterns were collected from the lactating cows in parity 2, in the middle of lactation, and daily milk yield was around 5 kg. After collection, raw milk was immediately frozen and pending analysis.

The TMR diets which were used by indoor feeding patterns consisted of maize silage, oat grass, cotton hulls and concentrate, which included maize, cotton protein, soybean meal, bran, molasses, minerals, vitamins, etc. The main composition of the grazed feeding pattern is maize straw, a mixture of maize and bran, oil dregs, oat grass and other grassland plants foraged freely during grazing. These samples were frozen (−20 °C) until used to determine the profile of volatile compounds and taste attributes.

### 2.2. HS-SPME/GC-MS

Volatile flavors were quantitated by HS-SPME/GC-MS. Thawed milk samples (10 mL) and internal standard were immediately incorporated in vials containing NaCl (1 g). 1 cm fiber coated with divinylbenzene/Carboxen/polydimethylsiloxane was used for extraction. The analysis of volatile compounds was carried out by Agilent 7890A gas chromatograph with 5975 mass spectrometry detection and, additionally, separated with a DB-WAX UI column (30 m × 0.25 mm, 0.25 µm). The initial temperature was held at 30 °C for 6 min, then increased at a rate of 10 °C/min to 240 °C and finally held for 3 min. Scan was used as the data acquisition mode. Volatile flavor compounds were identified by comparison with MS from a library database NIST.

### 2.3. E-Nose

The overall odor characteristics were determined by PEN3 (Win Muster Airsense Analytics Inc., Schwerin, Germany), comprising 10 sensors. When volatile flavor compounds pass through the instrument, the “odor fingerprint” can be detected by the sensors. The main groups and the corresponding performance description are listed in Table 1, as previously described by Chi, et al., [8]. Raw milk was placed in a dedicated vial with sodium chloride, capped, incubated for 300 s at 40 °C, and stirred constantly before being injected. The data acquisition time was 120 s.

### 2.4. E-Tongue

The taste properties of samples were assessed by SA 402B (Intelligent Sensor Technology Co., Ltd., Atsugi, Japan), which has 6 sensors that indicated 9 taste attributes, including bitterness, sourness, umami, saltiness, astringency, sweetness, aftertaste-bitterness, aftertaste-astringency and richness (aftertaste-umami). The taste attribute values were relative outputs using artificial saliva (e-tongue reference solution) as a standard, which test simulates the state of the human mouth when only saliva was present. Sensors and reference electrodes were pre-activated for at least 24 h. The activation of the sensor for sweetness and the detection of the sample were independent. Samples were appropriately diluted and filtered at room temperature. Each sample was assayed three times.

### 2.5. Statistical Analysis

The qualitative analysis of volatile organic compounds was carried out using MS combined with the retention index. The NIST 14 database was used for MS to identify unknown compounds. The compound’s retention index was determined by measuring the retention time of C7-C40 n-alkanes. The concentration of volatile organic compounds was estimated semi-quantitatively using the internal standard. An independent samples *t*-test (*p* < 0.05) was performed using SPSS 22.0. Principal component analysis (PCA), Partial Least Squares Discriminant Analysis (PLS-DA) and orthogonal partial least squares-discriminant analysis (OPLS-DA) were performed using Metabo Analyst 4.0 (https://metaboanalyst.ca/) (accessed on 26 April 2023) and SIMCA 14.1. OmicStudio tools were used for correlation analysis (https://www.omicstudio.cn/tool) (accessed on 26 April 2023).

## 3. Results

### 3.1. E-Nose Analysis

**Figure 1:**
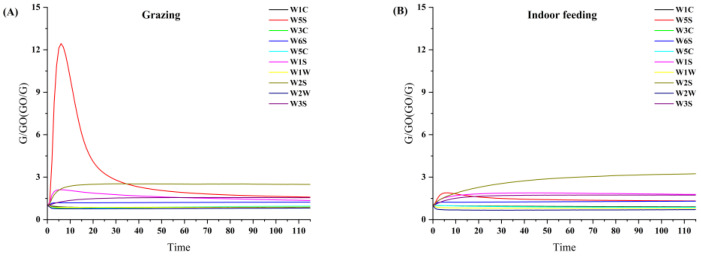
Response curve of raw milk samples under two feeding patterns. (**A**) Raw milk from grazing; (**B**) raw milk from indoor feeding.

Slight changes in aroma have resulted in the discrepancy between sensors’ responses [35]. According to the sensitive functional group corresponding to each sensor, the main characteristic odor composition of the sample can be inferred [17]. The response curves are presented in Figure 1. During enrichment, the initial response of the W5S sensor (red) 6 (which has a sensitivity to nitrogen oxides) was highest for both sets of samples, with grazing milk exceeding 12 and indoor feeding pattern raw milk below 2. As the enrichment time increases, the response of the W2S sensor (blue) (which has a sensitivity to alcohols, ketones and aldehydes) was highest during the final stabilization phase, with the indoor feeding group exceeding 3 and the grazing group below 3. The response of the W3S sensor (orange) was prominent (which has a sensitivity to long-chain alkanes), and the response was close to W1S.

**Figure 2:**
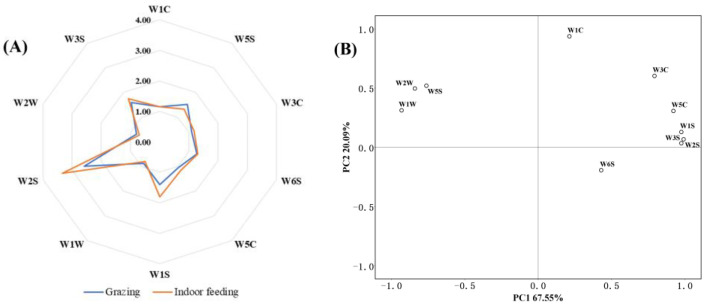
Electronic nose sensor evaluation results. (**A**) Radar chart of raw milk at different feeding patterns; (**B**) loading plot of principal component analysis of raw milk subjected to different feeding patterns.

The data of 110–115 s were converted into a radar map (Figure 2A). It was observed that the odor profile of raw cow’s milk between grazing and indoor feeding was significantly different. At the beginning of the detection, the highest enrichment was from the W5S sensor (red). The response of the W5S sensor was over 12 for raw milk in the grazing mode and below 2 for raw milk in the housed mode. Therefore, it was indicated that the grazing raw milk had a higher nitrogen oxide. As the enrichment time increased, the W2S sensor (light blue) reached the highest response and continued to grow, eventually plateauing at a high level. These results were similar to the GC-MS test; the raw milk from the grazing pattern has a high abundance of nitrogen oxides. The response values of W1W and W2W sensors less than 1 indicated a lower function [35,36]. In addition, the response value of most sensors under grazing was low in the whole test process, except the W5S sensor. The PCA plot explained 88% of the variances (Figure 2B). These results showed that the aroma profile was well represented by sensors of the e-nose.

**Figure 3:**
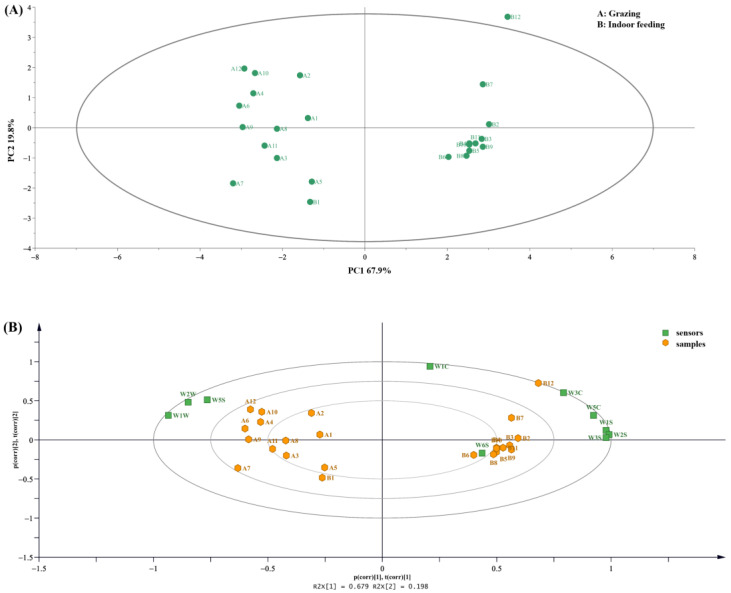
Principal component analysis based on the e-nose data set of samples. (**A**) Score scatter plot of raw milk at different feeding patterns; (**B**) biplot of raw milk at different feeding patterns.

The score scatter plot showed that samples from Holstein cattle reared intensively in an indoor feeding pattern were more consistent and clustered closely, while the distribution of raw milk in grazing were more dispersed (Figure 3A). The biplot (Figure 3B), as an overlay of the score and load plots, was also straightforward in terms of seeing the correlation with the sensors. The samples in group A were grazing feed, and the main sensors associated were the W2W sensor, W1W sensor and W5S sensor, while the W6S sensor was the closest to the housed samples, indicating that for housed cows, their raw milk was the most sensitive to this sensor.

### 3.2. E-Tongue Analysis

The reference solution consists of KCL and tartaric acid, so the tasteless point was −13 for sour tastes and −6 for salty tastes, and it was used as a benchmark. When the value is below, it means that the sample has no taste. An independent sample *t*-test was carried out for each taste attribute value to determine significant differences, whereas a *p* < 0.05 value was regarded as significant. Two groups showed significant correlations in all flavor attributes, except for sourness and umami (Table 2). Potentiometric difference between each sensitive electrode and the Ag/AgCl reference electrode in the equilibrium state was recorded as the response signal. The potential change of the sample was recorded and transformed into taste data by these sensors using set conversion factors.

**Figure 4:**
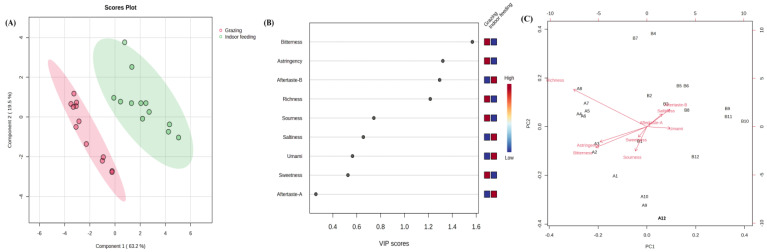
Partial Least Squares-Discriminant Analysis (PLS-DA) results based on electronic tongue. (**A**) Loading plot of raw milk under two feeding patterns. (**B**) VIP scores of raw milk based on electronic tongue under two feeding patterns, Each point in the graph represents the score of the VIP. There are corresponding numbers in the horizontal coordinates. (**C**) Biplot of raw milk in two feeding patterns.

The results of the e-tongue can be observed on the load diagram (Figure 4A). The two types of feeding practices had a more pronounced effect on the taste of raw milk. Two groups were clustered into one category and clearly differentiated, which was consistent with the electronic nose. The PLS-DA model was used for the discriminant analysis of taste attributes. The contribution rate of the two principal components was 82.7%. Therefore, it was indicated that the model has good discriminative and predictive ability. Raw milk samples were clearly separated into different areas with different feeding patterns. Through the PLS-DA model, four taste attributes with VIP > 1 were screened, which indicated that bitterness, astringency, aftertaste-B and richness had great differences in raw milk with different feeding patterns (Figure 4B). The distance represented the degree of contribution in the biplot plots (Figure 4C). From the distribution of taste attributes of the samples in the figure, the taste of raw milk in grazing mode was mainly concentrated in richness, astringency, bitterness, sweetness and sourness. In contrast, the taste of raw milk in the indoor feeding mode was mainly concentrated in salty, umami and bitter aftertaste and aftertaste astringency.

### 3.3. Volatile Analysis

Volatile flavor compounds of raw milk by GC-MS revealed 20 compounds (Table 3). These included 1 aldehyde (furfural), 1 ketone (2-nonanone), 5 alcohols (3,4-dimethylpentanol, 1-octen-3-ol, 2-furanmethanol, 2-ethyl-1-hexanol and 1-heptanol), 11 acids (acetic acid, butanoic acid, pentanoic acid, hexanoic acid, heptanoic acid, octanoic acid, dodecanoic acid, octadecanoic acid, nonanoic acid, n-decanoic acid and 9-decenoic acid), 1 ester (ethyl hexanoate), and 1 nitrogenous (oxime-, methoxy-phenyl-).

**Figure 5:**
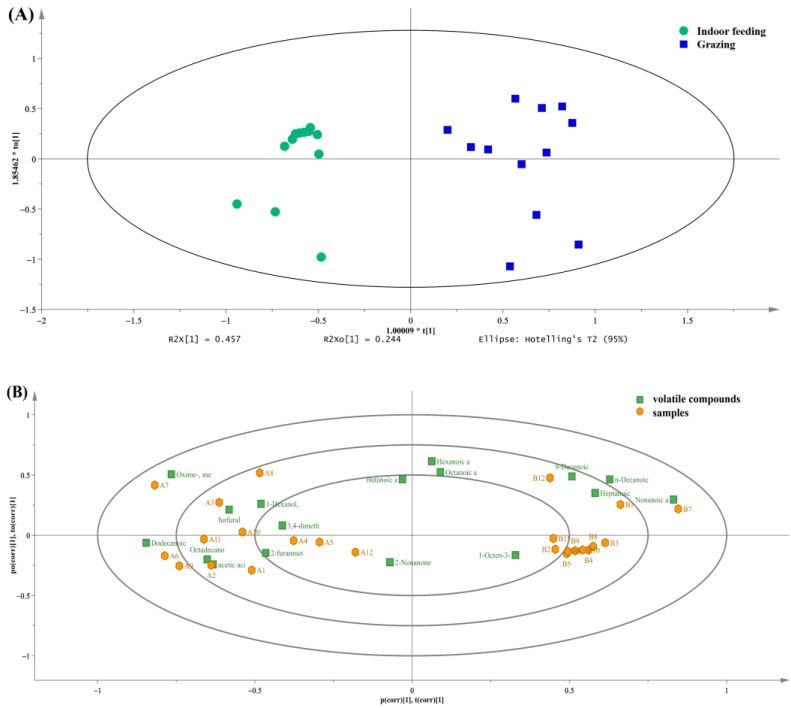
Orthogonal partial least squares discriminant analysis (OPLS-DA) results based on volatile flavor compounds data. (**A**) Score scatter plot of raw milk at different feeding patterns; (**B**) Biplot of raw milk at different feeding patterns.

OPLS-DA can filter out the between-group differences more accurately. The direction of the horizontal coordinate showed the differences in groups. The within-group differences have been observed on the vertical coordinate. In terms of the distribution of samples (Figure 5A), within-group variation was more pronounced for raw milk collected in the grazing mode. The arrangement of the samples was more dispersed, indicating that their diet had some inconsistency in the free-feeding state. In contrast to raw milk obtained in the indoor feeding pattern, which has a more concentrated distribution, raw milk obtained through an outdoor feeding pattern may be similar in terms of volatile flavor and behave more consistently.

Biplot overlapped the score and loading plots (Figure 5B). The distribution distances of the compounds and samples reflected their correlation, including 3,4-dimethylpentanol, acetic acid, furfural, hexanol, 2-furanmethanol, methoxyphenyl oxime, undecanoic acid and dodecanoic acid. The closer proximity of these compounds to the grazing samples may represent that they are unique to grazing as distinct components. While for raw milk samples obtained in the indoor feeding pattern, the abundance of 1-octen-3-ol, heptanoic acid, nonanoic acid, n-decanoic acid, 9-decenoic acid, butanoic acid, octanoic acid, hexanoic acid and 2-nonanone were likely to be higher.

**Figure 6:**
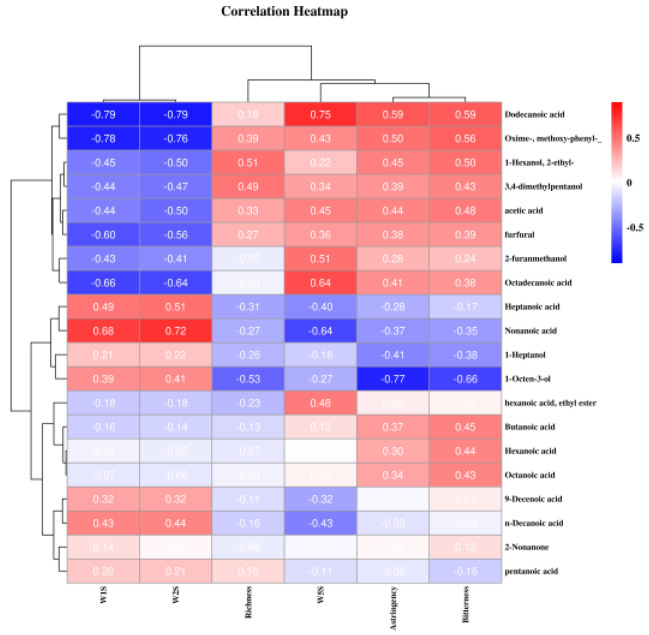
Correlation between volatile compounds and main intelligent sensory signals.

The correlation between volatile compounds and main intelligent sensory signals can be seen in Figure 6. Similar studies have also been conducted in pine mushrooms [35], cocoa bean shells [37] and pine nuts. Volatile flavor compounds were plotted on the Y axis, while the X axis had the main sensors of the electronic nose and key taste attributes of the electronic tongue. Most compounds were significantly correlated with the key sensors from the e-nose and the e-tongue. The contributions of both W1S and W2S sensors to sample differentiation were consistent, and they were negatively correlated to dodecanoic acid, Oxime-, methoxy-phenyl-, furfural, octadecanoic acid and nonanoic. By contrast, the response of the W5S sensor was positively correlated with dodecanoic acid and octadecanoic acid, but negatively correlated with nonanoic acid. The correlation results between the main taste properties of electronic tongue and volatiles were as follows: bitterness and astringency were positively correlated with dodecanoic acid, 1-hexanol, 2-pentanol and 3,4-dimethylpentanol; all three taste properties were negatively correlated with 1-octen-3-ol. These results showed that intelligent sensory devices were similar to the GC–MS. Moreover, it indicated that the e-nose could discriminate grazing and indoor feeding pattern by responding specifically to volatile compounds from raw milk. The correlation of intelligent sensory technology and the main volatile flavor compounds could further distinguish the samples and explain the difference between these methods. It could be critical for identifying or tracing raw milk from different feeding patterns.

## 4. Discussion

The purpose of this study was to explain flavor differences under different feeding patterns. The odor summarized profile was assessed by the electronic nose. The taste score was evaluated by the electronic tongue, and volatile flavor compounds were detected by HS-SPME/GC-MS. The results demonstrate that the ability of volatile profiling and intelligence sensory techniques can distinguish raw milk produced from grazing versus indoor feeding systems.

The response of the W5S sensor in the grazing pattern far exceeded that in the pasture-fed mode. According to the sensitivity of the sensors, we hypothesized that the grazing pattern has a higher abundance of nitrogen oxide in raw milk. GC-MS results confirmed that oxime, methoxy-phenyl- was the characteristic component detected in the grazing group. Oxime-, methoxy-phenyl- was once detected in yogurt, ultra-pasteurized milk and cheese [38,39]. It also has been reported to be a characteristic component of mushrooms and mountain plants [35]. Furthermore, the W5S sensor response was positively correlated with the levels of dodecanoic and octadecanoic acids. Consistent with previous studies that compare with a confinement system, farmers’ bulk milk samples of grazing pattern contained more octadecanoic acid and dodecanoic acid [40].

2-ethyl-1-hexanol has a “chemical/cleaning agent” aroma, which was detected in raw milk over refrigerated storage time and might be due to the packaging and refrigeration environment [41]. 1-Octen-3-ol was the common flavor active substance in milk and dairy products. The odor has a low threshold, which was often described as mushroom and grass. GC-MS results showed that the abundance of 1-octen-3-ol was positively correlated with the response of the W2S sensor, which may be due to the high content of silage in the forage on the farm.

Aldehydes could provide significant aromas due to their lower odor threshold, either pleasant or rancid [38]. W1S was thought to be more associated with volatiles containing methyl. The response values of W2S and W1S sensors were negatively correlated for 2-furanmethanol and furfural. 2-Furanmethanol and furfural were typical Maillard reaction products and have been observed in formula and heated milk, introducing caramel flavor and richness [42]. The high abundance of aldehydes may be the direct or indirect cause of sweetness in raw milk from grazing feeding patterns.

By means of an algorithm inherent to the electronic tongue, we obtained scores for the taste attributes in raw milk. The VIP scores, which were based on the PLS-DA model, indicated that bitterness, astringency, bitter aftertaste and richness represented the main taste differences. Compared to grass silage, maize gave a higher sweet odor and less boiled milk, saltness and metallic flavor [43]. Consistent with our research, Holstein cows on concentrated pasture, which have a high proportion of silage in their feed, scored higher for freshness and saltiness in raw milk. On the other hand, cows in the grazing mode, where the forage was dominated by maize straw, had a more prominent raw milk performance in terms of bitterness, richness and sweetness.

In our research, most of the volatile fatty acids are high in indoor feeding, with little or no detection in raw milk from the grazing pattern. In previous studies, large amounts of ethyl hexanoate were detected in cheese made from milk obtained from grazing cows, but no acetic acid was detected [21]. In this study, the bitterness and astringency of raw milk were positively correlated with the abundance of acetic acid. Hexanoic acid had been detected in raw milk from pasture, and ethyl hexanoate was detected from grazed feeding patterns, with the ethyl ester having a low odor threshold and possibly contributing a significant odor. However, the correlation between ethyl hexanoate and taste was not significant. The same conclusion has occurred in cheese [44]. Acids may result from lipolysis, lactose and degradation of amino acids, which were responsible for the rancid and bitter taste in milk [38,39]. Acid was the most dominant volatile substance category in milk and was thought to be associated with diet-related levels [30]. In general, long-chain fatty acids played a small role in flavor based on their high perceived threshold [45].

Ketones originated from the degradation of amino acids, which was an important component of milk products. High levels of ketones may have an adverse effect on flavor. 2-Nonanone was one of the characteristic components in the indoor feeding pattern. Silage has a limited effect on aldehydes, whereas ketones were more influenced by diet and ripening [38,46].

Based on the correlation between volatile compounds and main intelligent sensory signals, a combination of methods was used to judge the organoleptic quality of raw milk from different feeding patterns, and the expected results were achieved. Different dietary conditions may be the key factor for odor profiles. Some aromatics, such as oxime-, methoxy-phenyl-, octadecanoic acid, furfural and dodecanoic acid, were the critical volatile compounds in raw milk of grazing. Meanwhile, raw milk of indoor feeding pattern was unique in 2-nonanone, heptanoic acid and n-decanoic acid. All three detection techniques were valid and feasible for differentiating raw milk in both feeding patterns, and the compounds were significantly correlated with the key sensors by Person correlation analysis. This study will contribute to tracking and monitoring animal feeding systems by metabolic sources of VOC in the future.

Experimental results showed that there was large variability in the odor and taste of raw milk under different feeding patterns. Additionally, the milk yield of cows was lower from grazing than from indoor feeding. Grazing-fed cows might use body reserves for milk production, which had an impact on milk composition and further led to differences in milk taste. We believed that there were seasonal variations in the diet of grazing animals. Therefore, milk flavor should be continuously monitored in subsequent studies to compensate for the limitations imposed by seasonal differences.

## 5. Conclusions

These findings suggest that the volatile characteristics of raw milk from indoor feeding and grazing patterns differ significantly. Dietary conditions of cows may be the crucial element for different aroma profiles. Grazing milk achieved a significantly higher intensity for bitterness, richness and sweetness. W1S, W2S and W5S sensors played a major role in differentiating feeding systems. Some aromatic components, which included oxime-, methoxy-phenyl-, octadecanoic acid, furfural and dodecanoic acid, were the key volatile compounds in raw milk of grazing. Meanwhile, the raw milk under the indoor feeding pattern was unique in 2-nonanone, heptanoic acid and n-Decanoic acid. The results of the volatile compounds, odor profile and taste assessment were consistent in distinguishing between raw milk from two different feeding patterns. It will provide a comprehensive and accurate analysis method to study the effect of feeding patterns on raw milk flavor, which could be important for tracing or identifying raw milk under different feeding patterns.

## Figures and Tables

**Table 1:** Metal oxide conductivity gas sensors of the e-nose.

NO.	Sensor	Performance Description (Sensitivity to)
1	W5S	nitrogen oxides
2	W1S	methyl
3	W2S	alcohols, ketones, and aldehydes
4	W3C	ammonia
5	W1C	benzene
6	W5C	short-chain aromatic compounds and olefin
7	W1W	sulfur compounds
8	W2W	organic sulfides
9	W6S	hydrogen
10	W3S	long-chain alkanes

**Table 2:** Taste attribute values of raw milk based on electronic tongue.

Group	Sourness	Bitterness	Astringency	Aftertaste-B	Aftertaste-A	Umami	Richness	Saltiness	Sweetness
Grazing	−39.76 ± 0.57	2.38 ± 0.52	−2.41 ± 0.342	−0.20 ± 0.17	0.13 ± 0.051	8.76 ± 0.60	12.87 ± 2.21	11.88 ± 0.28	12.57 ± 0.13
Indoor feeding	−40.86 ± 0.50	0.042 ± 1.332	−4.38 ± 1.30	1.73 ± 1.154	0.51 ± 0.35	9.60 ± 0.55	11.07 ± 1.80	12.85 ± 0.53	11.79 ± 0.39
*p* value	0.37	0.02	0.01	0.00	0.00	0.77	0.24	0.04	0.01

**Table 3:** Volatile compounds identified by HS-SPME/GC–MS.

No.	Compound	Retention Time	CAS#	Formula
1	Ethyl hexanoate	11.82	123-66-0	C_8_H_16_O_2_
2	3,4-Dimethylpentanol	13.78	64502-86-9	C_7_H_16_O
3	2-Nonanone	14.24	821-55-6	C_9_H_18_O
4	Acetic acid	15.09	68475-71-8	C_2_H_4_O_2_
5	1-Octen-3-ol	15.12	3191-86-4	C_8_H_16_O
6	Furfural	15.19	98-01-1	C_5_H_4_O_2_
7	1-Hexanol, 2-ethyl-	15.6	104-76-7	C_8_H_18_O
8	1-Heptanol	15.2	111-70-6	C_7_H_16_O
9	Butanoic acid	17.32	1977-33-9	C_10_H_19_NO_5_
10	Pentanoic acid	17.31	109-52-4	C_5_H_10_O_2_
11	2-Furanmethanol	17.83	98-00-0	C_5_H_6_O_2_
12	Oxime-, methoxy-phenyl-	18.81	1775-61-7	C_8_H_9_NO_2_
13	Hexanoic acid	19.79	142-62-1	C_6_H_12_O_2_
14	Heptanoic acid	20.92	1173022-17-7	C_7_H_14_O_2_
15	Octanoic acid	22	124-07-2	C_8_H_16_O_2_
16	Dodecanoic acid	24.01	143-07-7	C_12_H_24_O_2_
17	Octadecanoic acid	27.57	57-11-4	C_18_H_36_O_2_
18	Nonanoic acid	23.01	112-05-0	C_9_H_18_O_2_
19	n-Decanoic acid	24	334-48-5	C_10_H_20_O_2_
20	9-Decenoic acid	24.53	14436-32-9	C_10_H_18_O_2_

## Data Availability

Data is contained within the article.
